# Neuroinflammation and Oxidative Stress in the Pathogenesis of Autism Spectrum Disorder

**DOI:** 10.3390/ijms24065487

**Published:** 2023-03-13

**Authors:** Noriyoshi Usui, Hikaru Kobayashi, Shoichi Shimada

**Affiliations:** 1 Department of Neuroscience and Cell Biology, Graduate School of Medicine, Osaka University, Suita 565-0871, Japan; 2 United Graduate School of Child Development, Osaka University, Suita 565-0871, Japan; 3 Global Center for Medical Engineering and Informatics, Osaka University, Suita 565-0871, Japan; 4 Addiction Research Unit, Osaka Psychiatric Research Center, Osaka Psychiatric Medical Center, Osaka 541-8567, Japan; 5 SANKEN (Institute of Scientific and Industrial Research), Osaka University, Suita 567-0047, Japan

**Keywords:** autism spectrum disorder, maternal immune activation, viral infection, inflammation, oxidative stress, hydrogen, silicon, biomarker

## Abstract

Autism spectrum disorder (ASD) is a neurodevelopmental disorder (NDD) characterized by impairments in social communication, repetitive behaviors, restricted interests, and hyperesthesia/hypesthesia caused by genetic and/or environmental factors. In recent years, inflammation and oxidative stress have been implicated in the pathogenesis of ASD. In this review, we discuss the inflammation and oxidative stress in the pathophysiology of ASD, particularly focusing on maternal immune activation (MIA). MIA is a one of the common environmental risk factors for the onset of ASD during pregnancy. It induces an immune reaction in the pregnant mother’s body, resulting in further inflammation and oxidative stress in the placenta and fetal brain. These negative factors cause neurodevelopmental impairments in the developing fetal brain and subsequently cause behavioral symptoms in the offspring. In addition, we also discuss the effects of anti-inflammatory drugs and antioxidants in basic studies on animals and clinical studies of ASD. Our review provides the latest findings and new insights into the involvements of inflammation and oxidative stress in the pathogenesis of ASD.

## 1. Introduction

Autism spectrum disorder (ASD) is a heterogeneous neurodevelopmental disorder (NDD) that causes pervasive abnormalities in social communication, repetitive behaviors, and restricted interests [1,2]. The etiology of ASD is thought to involve complex, multigenic interactions and possible environmental factors [2]. The prevalence of ASD has been recently reported to be 1 in 44 (2.27%) patients in the US [3,4]. The male-to-female prevalence ratio of ASD has been reported as 4:1 [5]. However, no effective treatment for ASD has been developed to date. Although early interventions for ASD have been reported to improve symptoms [6,7,8], these interventions are based on symptomatic treatments, such as cognitive behavioral therapy. Thus, the development of early treatment and diagnosis methods has gained importance in the management of ASD.

In the prenatal environment, maternal immune activation (MIA), stress, drug exposure, and undernutrition are well-known environmental factors that affect offspring health [9,10,11,12,13,14]. These environmental factors are highly associated with various NDDs and psychiatric disorders, including ASD, attention-deficit/hyperactivity disorder (ADHD), schizophrenia, and depression [9,10,11,12,13,14]. MIA is also associated in the induction of oxidative stress involved in the pathogenesis of ASD [15,16]. Oxidative stress is a phenomenon that damages cells and other organs by producing free radicals and peroxides due to a disorder of the redox state [17,18,19,20,21,22]. A meta-analysis study reported that maternal infection during pregnancy was associated with a 12% increased risk of ASD in offspring [23]. In addition, another meta-analysis study has reported that preventing or safely treating maternal infections could reduce the incidence of ASD by 12% to 17% [24]. Moreover, the infection of coronavirus disease 2019 (COVID-19) during pregnancy is associated with MIA and can result in the risk of ASD onset by alteration of maternal and fetal immune responses [9,11,25,26]. Therefore, a thorough understanding of the role of inflammation and oxidative stress in the pathogenesis of ASD has also gained significance.

In this review, we first focus on environmental changes in fetal brain development caused by inflammation and oxidative stress and their roles in the pathogenesis of ASD. We next discuss inflammation and oxidative stress in the pathogenesis of ASD, and discuss the studies using anti-inflammatory drugs and antioxidants as potential prophylactic and therapeutic agents for ASD in basic and clinical research. In addition, we discuss the potential of hydrogen medicine, including our newly developed Si-based hydrogen-producing agent that continuously and effectively produces hydrogen in the body [27,28,29]. We have demonstrated that Si-based agent can prevent inflammation and oxidative stress in mouse models of maternal-fetal transmission and ASD [30,31]. The prevention and treatment of neuroinflammation and oxidative stress in the pathogenesis of ASD may be effective in preventing the onset of ASD and health risks in fetuses and children.

## 2. MIA and Neuroinflammation in ASD

**Figure 1:**
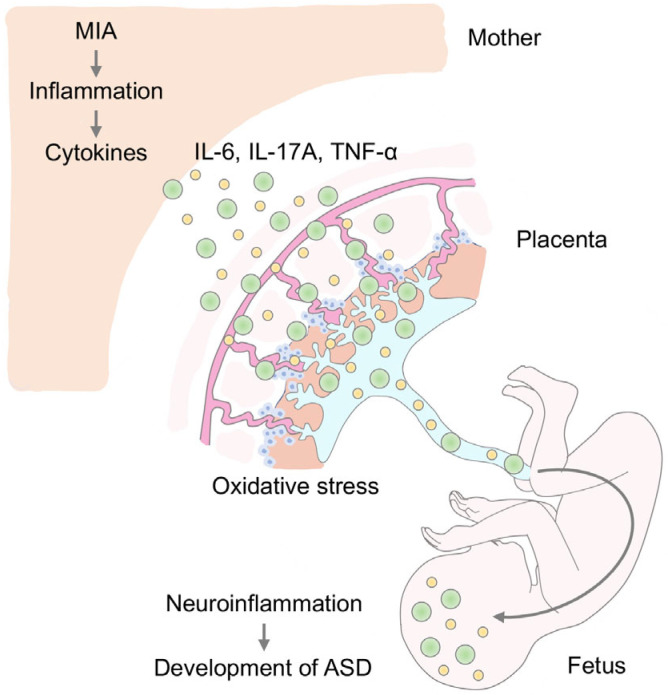
A model diagram of maternal-placental-fetal neuroinflammation in ASD pathogenesis. Maternal immune activation (MIA) induces immune response in the mother’s body against viral and microbial infections, autoimmune responses, and/or stress. MIA triggers inflammation and cytokines productions such as IL-6, IL-17A, and TNF-α. These cytokines damage placental structures and cross from placenta to fetus. Eventually, maternal and placental cytokines and oxidative stresses also damage the fetal brain and body themselves, resulting in neuroinflammation in the fetal brain. These events are potential risks to the development of ASD.

MIA is the most well-known environmental factor for the onset of ASD [9,10,11,12,13,14,32,33]. MIA is triggered by infection or autoimmune predisposition during pregnancy, and cytokines produced by MIA are transmitted through the placenta to the fetal brain (Figure 1), increasing the risk of onset of and vulnerability to developmental and psychiatric disorders such as ASD, intellectual disability (ID), major depressive disorder (MDD), bipolar disorder, and schizophrenia [9,10,11,12,34,35]. In addition, MIA causes vulnerability in the brain after birth and contributes to the future risk of diseases such as microcephaly, cerebral palsy, white matter lesions, and addiction [9,10,11]. MIA also increases the risk of stillbirth, miscarriage, and intrauterine growth retardation [9,11].

Many studies have reported inflammation as a risk factor for ASD [9,10,11,13,36]. Animal models of MIA use poly(I:C), a double-stranded RNA that mimics viral infections [37,38]. Poly(I:C) regulates inflammation and immunity by activating downstream signals through toll-like receptor 3 (TLR3) [39]. MIA triggers maternal inflammation, and Th17 cells activated by interleukin (IL)-6 secrete IL-17A in mice [38,40]. Inflammation also occurs in the placenta, which connects the mother and fetus, and IL-17A mRNA expression is upregulated in the placenta [38]. Maternally derived IL-17A crosses the placenta and induces neuronal cell death by acting on IL-17A receptors expressed in the embryonic mouse brain [38]. This cell death is thought to cause a reduction in the number of parvalbumin-positive GABAergic neurons in the mouse cortex [40], resulting in a disturbance in the E/I balance and leading to ASD pathogenesis [41]. MIA-induced neuroinflammation is one of the causes of ASD [11]. MIA-induced IL-17A expression impairs social behavior by causing neuronal cell death in the embryonic mouse brain [38], although IL-17A has also recently been reported to promote sociality in MIA mouse offspring [42]. In fact, a subset of children with ASD appear to show improvements in ASD symptoms during the course of fever, a sign of systemic inflammation [43,44]. 

One of the effects of MIA on brain development is the activation and accumulation of microglia [45,46,47]. Microglia express IL-17A receptors and respond to inflammation and immune responses in the mouse brain [48]. Direct administration of IL-17A into the lateral ventricle of mouse fetuses to investigate the effects of MIA on microglia has been shown to induce the activation of microglia and changes in their localization to the paraventricular [46]. Furthermore, a recent study reported a long-term decline in microglial immunoreactivity in the MIA mouse offspring [49].

MIA is also known to dysregulate ASD-associated and neurodevelopmental genes [50,51,52,53]. For example, MIA downregulates the genes involved in axonal guidance, neurogenesis, and the cytoskeleton, whereas it upregulates the genes involved in translation, cell cycle, and DNA damage [50]. MIA also induces ASD-like behavioral abnormalities such as anxiety-like, repetitive, and sensorimotor gating behaviors with the volume changes of the dorsal and ventral hippocampus and anterior cingulate cortex in MIA mouse offspring [51]. Recent single-cell transcriptome analyses showed that the genes in the mouse embryonic brain affected by MIA include those involved in mRNA translation, ribosome biogenesis, and stress signals, resulting in reduced global mRNA translation and altered nascent proteome synthesis [53]. 

Lipopolysaccharide (LPS) is a molecule composed of lipids and carbohydrates found on the cell wall surface of Gram-negative bacteria such as *Escherichia coli*, and mimics bacterial infection to induce inflammation [54]. LPS is also used a reagent for making animal models of MIA [55], and it regulates inflammation and immunity by activating downstream signals through toll-like receptor 4 (TLR4) [39,54]. Administration of either LPS or poly(I:C) are commonly used methods to induce MIA, and the phenotype of ASD model animals obtained by both is similar [56]. Interestingly, sex-specific vulnerabilities in the placenta and brain have been reported in mice after prenatal immune disruption by mild MIA induced by lipopolysaccharide (LPS) [57]. A previous study has reported that LPS-induced MIA causes acute vascular damage in the placenta, along with transient hypoxia and reduced neural progenitor cell proliferation in the developing embryonic mouse brain [58]. In addition, reduced numbers of SATB2-, PV-, and TBR1-positive cells have also been reported in the cortex of LPS-induced MIA offspring, indicating abnormalities in the cortical lamina and connectivity in MIA mouse offspring [58]. 

Regarding sex differences in the effects of MIA model mice, severe necrosis-related placental damage, elevated placental inflammation, elevated cortical hypoxia, social impairment, increased repetitive behaviors, and impaired working memory have been observed in male offspring [57], while mild placental damage, moderate placental inflammation, moderate cortical hypoxia, and anxiety-like behaviors have been observed in female offspring [57]. These sex-related differences in the influence of MIA may be linked to the epidemiological characteristics of the male-to-female ratio of ASD. In addition, MIA studies using model animals have reported that MIA predominantly affects more males than females [33]. For instance, these animal studies found more impairments in males than in females for repetitive behaviors [59,60], motor development with reduced numbers of Purkinje cells in the cerebellum [61], and learning and memory with hippocampal dysfunction [62,63]. These findings using animal models are reflected in the male-to-female prevalence ratio of ASD as 4:1 in human [33]. This is an interesting finding that there are sex differences not only in genetic factors but also in environmental factors [57]. Males appear to be more vulnerable to MIA, however the mechanisms underlying this association remain unclear, thus it is required that future studies in this field uncover the mechanism.

In MIA, the placenta is one important factor to understand the pathogenesis of ASD. As mentioned above, the mouse placenta is damaged by inflammatory cytokines induced by MIA [30,57]. Inflammation causes placental necrosis and loss of growth potential [30,57]. Depending on the severity of placental impairment, it can cause stillbirth or miscarriage [10,11,30]. Even if it is a tolerable impairment, the dysfunction of the placenta causes serious damage to the fetal development, such as impairment of the placental barrier and poor nutritional supply to the fetus [10,11]. In particular, when the placental barrier, which plays the role of the blood-brain barrier, is damaged by MIA, blood components derived from the mother flow directly into the fetus [16,64,65]. Such maternal components include inflammatory cytokines produced, oxidative stress, and antibodies against these by MIA [33,66,67,68]. In the fetus, the blood-brain barrier has not yet matured [69,70], and blood supplied to the fetus by the mother through the placenta flows directly into the fetal brain. In addition, inflammatory cytokines not only inhibit the development of the fetal brain, but also cause serious damage to the fetal brain by obstructing it [16,64]. Through such processes, MIA is thought to contribute to the pathogenesis of ASD [10,11].

On the other hand, previous studies reported increased inflammation in children with ASD [9,11,64], and it is thought to lead to the formation of pathological conditions or the aggravation of pathological conditions underlying ASD [11,64,65]. For example, inflammatory cytokines such as transforming growth factor-β1 (TGF-β1), hepatocyte growth factor (HGF), epidermal growth factor (EGF), platelet-derived growth factor-BB (PDGF-BB), and tumor necrosis factor-α (TNF-α) have been reported to be present in the peripheral serum and plasma of children with ASD [71,72,73,74,75]. It has also been reported that there are higher concentrations of IL-1β, IL-4, IL-6, IL-8, and IL-13 in the peripheral serum and plasma of children with ASD [16,76,77]. In addition, elevated inflammatory molecules such as IL-1β, IL-6, TNF, and monocyte chemoattractant protein-1 (MCP-1) were found in the postmortem brain and cerebrospinal fluid (CSF) in children with ASD [77,78,79,80,81]. Furthermore, IL-1β and IL-4 in neonatal blood samples have also been reported to be associated with ASD severity [82]. These studies demonstrate that a systemic inflammation is associated with the pathophysiology of children with ASD.

MIA-induced neuroinflammation, which is closely related to oxidative stress, is one of the causes of ASD [83]. Inflammation-induced cell death in the fetal brain and oxidative stress substantially influence fetal brain development [64,65]. However, many aspects related to the effects of neuroinflammation on brain development in the pathogenesis of ASD remain unclear. The involvement of neuroinflammation in the pathogenesis of NDDs has been discussed recently in both clinical and basic research fields [64,65], and neuroinflammation has been suggested to be important not only in ASD but also in other NDDs, such as ADHD [9,11]. Therefore, inhibition of inflammation within the first term in MIA is key to preventing the risk of ASD onset, highlighting the need for preventive drugs that protect both the mother and fetus from the influence of MIA safely and without side effects.

## 3. MIA and Oxidative Stress

Many findings have also been reported on the association between MIA and oxidative stress [15,16]. Damage to the mouse embryos and placenta by MIA includes hypoxia, oxidation, stress, and cell death [57,84]. The placenta is a site of reactive oxygen species (ROS) metabolism that sustainably generates oxidative stress, and the placenta produces antioxidants to keep oxidative stress under control [85]. MIA-affected placental histopathology and oxidative stress such as cyclooxygenase-2 (COX-2) and malondialdehyde can impair nutritional supply from the mother and placenta to the fetus in mice [84,86]. 

MIA has been reported to induce the expression of the ROS-producing enzyme NADPH oxidase 1 (Nox1) in the fetal brain, leading to ASD-like behaviors and reductions of TBR1-positive layer 6 neurons in the mouse cortex and Purkinje cells in the mouse cerebellum [87]. LPS-induced MIA was also reported to selectively alter the expression profiles of genes related to neuronal migration (*Dlx1* and *Dlx2*) and oxidative stress (*Apold1, Ahsp,* and *Bnip3*) in mice [84]. In addition, increased neuronal and glial densities with ultrastructural features reflecting increased neuroinflammation and oxidative stress have been reported in female mouse offspring exposed to MIA [88]. 

It is well-known that the impairment of mitochondria is associated with pathophysiology of ASD, however it is not well-known that mitochondrial dysfunction is also caused by MIA [15]. The mitochondria act as mediators and initiators of inflammatory processes, and play essential roles in innate immunity and inflammatory responses [89]. In the brain development, mitochondria play roles in biological processes in neurogenesis, gliogenesis, synaptogenesis, and blood-brain barrier (BBB) development [15,90,91,92,93,94]. Previous studies have reported that MIA causes the changes in terms of structure, morphology, density, and functions in mitochondria of the brain of model animals [15,95,96]. MIA induces alterations in hippocampal morphology, particularly in synaptic ultrastructure, including mitochondria, with disruption of mitochondrial electron transport chain and reduction of mitochondrial membrane potential in rats [95]. MIA also causes increased oxidative stress levels such as ROS and decreased antioxidant levels within mitochondria in offspring animals [15,95]. A decreased energy dysfunction of mitochondria has also been reported in the cortical neurons of MIA rat offspring [96]. Interestingly, it is also reported that such mitochondria dysfunction is recovered with transplantation of normal mitochondria into the prefrontal cortex (PFC) of MIA rat offspring [96]. 

These studies demonstrate that MIA not only causes inflammation but also oxidative stress via mitochondrial dysfunction, which damages the mother, placenta, and fetus, resulting in the risk of ASD and neurodevelopmental abnormalities in the offspring. However, it should be noted that it has not been proven that the increased oxidative stress in MIA was directly induced by inflammation and may be a secondary aspect resulting from inflammation.

## 4. Oxidative Stress in ASD

**Figure 2:**
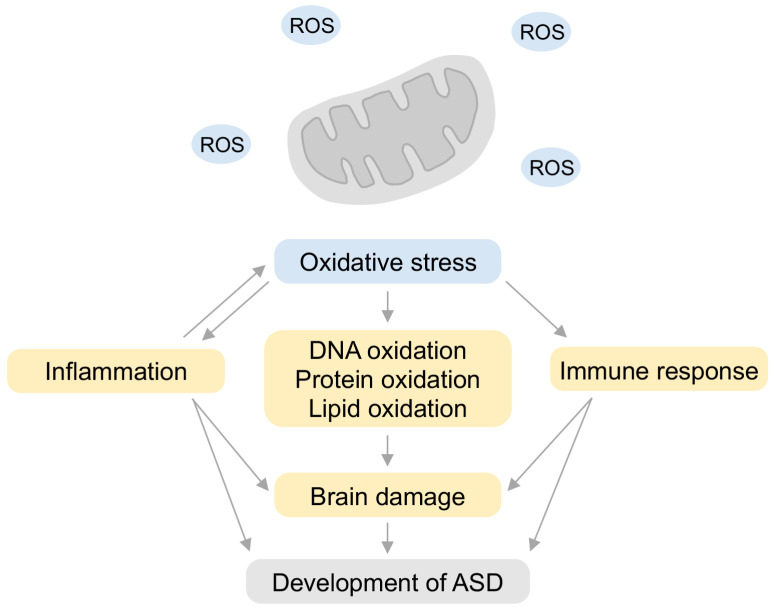
A model diagram of oxidative stress in ASD pathogenesis. Oxidative stress typified by mitochondrial disorders induces inflammation, and also oxidates DNA, protein as well as lipids. These oxidants are potential negative factors for damage of the brain and body. Subsequently, this damage and oxidative stress are risks of the development of ASD. ROS: reactive oxygen species.

Previous studies have reported that oxidative stress is involved in the pathogenesis of ASD [97,98,99,100]. In general, oxidative stress reflects the harmful effects of oxidative reactions in living organisms ROS reacts with biopolymers such as DNA, lipids, proteins, and enzymes in vivo to induce lipid peroxidation, gene mutation, protein denaturation, and enzyme inactivation [17,18,19,20,21,22] (Figure 2). Increased oxidative stress is thought to increase biological oxidative damage at the molecular level, leading to various diseases and accelerated aging [21,22,101,102,103]. 

Oxidative stress in children with ASD has been reported by the mitochondrial dysfunction and increased oxidative stress markers such as decreased antioxidant enzyme activity, increased lipid peroxidation, and accumulation of advanced glycation products in peripheral blood [97,99,100,104,105,106,107,108] (Figure 2). Mitochondria play important roles in free radical generation, ATP formation, and apoptosis [15]. Mitochondrial dysfunction has been reported in patients with ASD [108,109,110] and is considered one of the causes of ASD pathology. In fact, oxidative stress is increased due to abnormalities in the mitochondrial electron transport system in the brains of patients with ASD [111]. Such increased oxidative stress due to mitochondrial dysfunction has been reported in several studies, indicating its strong association with ASD pathology [97,99,100,112,113]. Inactivation of mitochondrial aconitase, a tricarboxylic acid (TCA) cycle enzyme that catalyzes the conversion of citrate to isocitrate, has been reported in children with ASD [99], and lipid peroxidation markers are also elevated in patients with ASD [100]. The levels of antioxidant serum proteins, transferrin (iron-binding protein), and ceruloplasmin (copper-binding protein) are decreased in children with ASD [107]. Ceruloplasmin inhibits the peroxidation of membrane lipids catalyzed by metal ions such as iron and copper [100,114]. Transferrin acts as an antioxidant by reducing the concentration of free ferrous ion, and abnormalities in ceruloplasmin and transferrin levels may lead to abnormal iron and copper metabolism in patients with ASD [100,115]. Previous case-control studies have reported that children with ASD show abnormal plasma metabolite levels in the glutathione redox pathway [116,117,118]. A decrease in reduced glutathione (GSH), increase in oxidized glutathione disulfide (GSSG), and decrease in the glutathione redox ratio (GSH/GSSG) have been reported in children with ASD [116].

Although there are a lot of studies on oxidative stress and glutathione as blood biomarkers in children with ASD [99,119,120,121,122,123], the presence of oxidative stress and glutathione in the postmortem brain has not been well studied. In postmortem assessments of children with ASD, increased levels of 3-nitrotyrosine (3-NT), an oxidative protein damage marker, have been reported in the cerebellum [119]. Glutathione redox imbalance and increased biomarkers of oxidative stress have also been reported in the cerebellum and Brodmann area 22 (BA22) in children with ASD [99]. In addition, 8-hydroxydeoxyguanosine (8-OHdG) levels were increased and associated with reductions in GSH and GSH/GSSG in the temporal cortex and cerebellum in children with ASD [99]. Increased oxidative protein levels and DNA damage have been reported to be associated with reduced plasma glutathione (GSH)/oxidized glutathione (GSSG) levels in children with ASD [120], demonstrating decreased GSH/GSSG redox/antioxidant capacity and increased oxidative stress in ASD.

Our previous study also reported increased oxidative stress in children with ASD [121,122,123]. Using lipidomics analyses, we identified 48 metabolites in ASD as novel targets involved in lipid biosynthesis and metabolism, oxidative stress, and synaptic function [121]. Consistent with previous studies that showed GSH reduction and oxidative stress in ASD [83,99,100,107,117,120,124], we found that a significant reduction in cysteine glutathione disulfide in children with ASD was involved in the downregulation of cysteine metabolism and oxidative stress [121]. In general, the major antioxidants GSH and cysteine are responsible for neutralizing the damage caused by oxidative stress [125,126]. GSH is a major cellular radical scavenger that plays an important role in protecting cells from exogenous and endogenous toxins, particularly in the brain [97,124,127]. Decreased GSH levels have been reported in several human ASD studies, and are associated with increased oxidative stress and decreased detoxification capacity [97,99,124,127]. On the other hand, our previous findings demonstrated that oxidative stress in children with ASD is not simply increased but shifted in a complicated pattern involving multiple types of ROS and antioxidants [122]. Decreased protection against hydroxyl radicals may be the fundamental mechanism underlying these changes.

These studies demonstrate the mechanisms linking oxidative stress with lipid abnormalities, inflammation, aberrant immune responses, impaired energy metabolism and excitotoxicity, glutathione redox imbalance, and oxidative stress in the pathogenesis of ASD. The mammalian brain is particularly exposed to more oxidative stress than other organs because it accounts for approximately 20% of the basal oxygen consumption, but only a small percentage of the body weight. This difference is even more pronounced in children who have smaller bodies, but not very small brains [97].

## 5. Relationship between Inflammation and Oxidative Stress

As mentioned in the previous sections, inflammation and oxidative stress are closely related [128,129]. Inflammatory processes induce oxidative stress and mitochondrial dysfunction, which can exacerbate oxidative stress and trigger negative feedback that leads to downstream abnormalities in brain development and its dysfunction, leading to the pathogenesis of neurodevelopmental disorders such as ASD and ADHD [16,128,129]. Inflammation and oxidative stress are spectrums and both are essential for normal physiological functions of living organisms such as immunity, while excessive inflammation and oxidative stress are also major causes of cell and tissue damage [125,126,130,131]. Both factors are important for understanding the pathogenesis of ASD and potential therapeutic targets [65,98,129,132].

## 6. Anti-Inflammatory Strategy Targeting Inflammation in ASD

**Figure 3:**
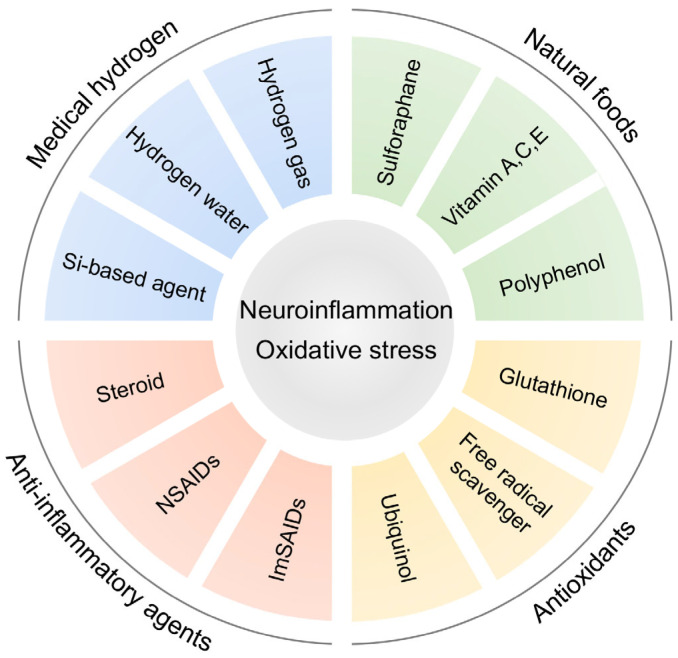
Neuroinflammation and oxidative stress as potential therapeutic targets in ASD pathogenesis. Representative anti-inflammatory agents and antioxidants for preventing neuroinflammation and oxidative stress. NSAIDs: non-steroidal anti-inflammatory drugs, ImSAIDs: Immune selective anti-inflammatory derivatives.

Understanding inflammation in the pathophysiology of ASD may provide new insights into therapeutic strategies for ASD (Table 1 and Table 2). Interactions among immune, nervous, and endocrine systems are important in inflammation responses [15,65,129,132]. Commonly used drugs for inflammation include steroid, non-steroidal anti-inflammatory drugs (NSAIDs), and immune selective anti-inflammatory derivatives (ImSAIDs) (Figure 3). Corticosteroids, a class of steroids, are anti-inflammatory drugs prescribed for a variety of conditions [133,134]. Inflammatory markers such as IL-6 and TNF-α are elevated in patients with ASD, schizophrenia, and depression [16,76,77,78,79,80,81,135,136,137]. Systemic corticosteroids have potent anti-inflammatory and immunosuppressive properties, but increased risks of severity of symptoms and recurrence in these disorders have also been reported [138]. NSAIDs are therapeutic agents that reduce inflammation, pain, and fever, and prevent blood clots [139,140]. Aspirin, ibuprofen, and naproxen are well-known NSAIDs. NSAIDs work by inhibiting the activity of cyclooxygenase enzymes such as COX-1 and COX-2, which are involved in the synthesis of prostaglandins, responsible for inflammation, and thromboxane, responsible for blood clotting [139,140]. ImSAIDs are unrelated to steroid hormones and are categorized of peptides with anti-inflammatory properties such as phenylalanine-glutamate-glycine (FEG) and tripeptide derived from submandibular gland peptide-T [141,142]. ImSAIDs act on the activation and migration of inflammatory cells such as neutrophils in the immune system that amplify the inflammatory response [141,142]. This pathway of immune-endocrine transmission is the cervical sympathetic trunk-submandibular gland (CST-SMG) axis [143].

In animal models of ASD, antibody therapy that directly targets inflammatory cytokines produced by MIA and ASD pathology are being investigated [38]. In MIA model mice, ASD-like behaviors such as impairment of social behaviors and repetitive behaviors and morphological abnormalities in the cortex were rescued by anti-IL-17a antibody injection directly into the fetal ventricle against maternal IL-17a [38]. Similarly, rescue of ASD-like phenotypes by maternal injection of anti-IL-6 antibodies has been reported in mouse models of ASD [144,145]. In addition, phenotypic improvement in ASD-like behaviors by the ketogenic diet has been reported in MIA mouse offspring [146]. A ketogenic diet has also been reported to have neuroprotective effects in neurodegeneration [147,148]. Moreover, oral probiotics containing bifidobacteria and lactobacilli administration to pregnant MIA mice reduced IL-6 and IL-17a inflammatory cytokines and prevented ASD-like behaviors and loss of GABAergic neurons in the PFC of MIA mouse offspring [149].

In the clinical studies, there are many studies on therapeutic strategies for ASD that target inflammation [11,16,150]. In a randomized, double-blind, placebo-controlled trial, the combination of risperidone and celecoxib, a COX-2 inhibitor was superior to risperidone alone in treating hypersensitivity, social withdrawal, and stereotypes in children with ASD [151]. In a double-blind, placebo-controlled crossover study, administration of neuropeptide ORG2766, a synthetic analog of adrenocorticotropic hormone (ACTH), to children with ASD increased the quantity and improved the quality of social interactions [152]. It is also reported that children with ASD treated with a dietary formulation containing the natural flavonoids luteolin and quercetin showed improvement in attention, social communication, and living skills [153]. In addition, a subgroup of children with ASD treated with luteolin and quercetin showed improvements of social communication and living skills, and decreases in IL-6 and TNF-α in the serum [79].

In contrast, previous studies have also reported that the use of NSAID during pregnancy is only associated with intellectual disability, not in ASD [154]. Corticosteroids have also been reported to increase the risk of severe ASD symptoms [138]. Therefore, therapeutic strategies targeting inflammation during pregnancy require careful discussion, basic research using animal models, and clinical studies.

**Table 1:** Summary of human studies for neuroinflammation and oxidative stress in ASD.

Subjects	Study	Sample Size	Treatments	Results	Reference
ASD children (4–12 years)	Randomized, double-blind, placebo-controlled trial	60	Combination of risperidone and COX-2 inhibitor, 10 weeks	Superior to risperidone alone in treating hypersensitivity, social withdrawal, and stereotypes	[152]
ASD children (mean age of 8.7 ± 2.7 years)	Double-blind, placebo-controlled crossover study	14	ORG2766, a synthetic analog of ACTH, 4 weeks	Improved quantity and quality of social interactions	[153]
ASD children (4–10 years)	Prospective, open-label trial	40	Flavonoids (luteolin and quercetin), 26 weeks	Improvements in attention, social communication, living skills	[154]
ASD children (4–10 years)	Prospective, open-label trial	38	Flavonoids (luteolin and quercetin), 26 weeks	Improvements in social communication and living skills, decreases in IL-6 and TNF-α in the serum	[79]
ASD children (6–19 years)	Double-blind, placebo-controlled study	18	Vitamin C, 10 weeks	Improved sensorimotor behaviors	[155]
ASD children (3–6 years)	Prospective, open-label trial	24	Ubiquinol, 3 months	Improved communication	[156]
ASD children (3–12 years)	Randomized, parallel, placebo-controlled study	90	Coenzyme Q10, 3 months	Reduced oxidative stress and ASD symptoms	[157]
ASD individuals (13–27 years)	Double-blind, placebo-controlled trial	29	Sulforaphane, 18 weeks	Improved social interaction and communication	[158]
ASD children (3.2–10.7 years)	Randomized, placebo-controlled trial	33	NAC, 12 weeks	Improvement in hypersensitivity subscales	[159]
ASD children (3.5–16 years)	Randomized, double-blind, placebo-controlled trial	47	Combination of risperidone and NAC, 8 weeks	Reduced hypersensitivity subscales	[160]
ASD children (4–12 years)	Randomized, double-blind, placebo-controlled clinical trial	40	Combination of risperidone and NAC, 10 weeks	Reduced hypersensitivity	[161]
ASD children (mean age of 10.9 ± 3.9 years)	Pilot feasibility study	16	Cocoa, 4 weeks	Improvements in social communication, erratic behavior, self-regulatory behavior	[162]

ACTH: adrenocorticotropic hormone, NAC: N-acetyl L-cysteine.

**Table 2:** Summary of animal studies for neuroinflammation and oxidative stress in ASD.

Models	Sample Size	Treatments	Results	Reference
MIA mice, 20 mg/kg poly(I:C) at E12.5	*n* = 25–38, analyzed at E18.5 or young adults	Anti-IL-17a antibody at E14.5	Reduced ASD-like behaviors and morphological abnormalities in cortex	[38]
MIA mice, 2.5 mg/kg poly(I:C) at E12 to E16	*n* = 10, analyzed at young adults	Anti-IL-6 or IL-1β antibodies at E12-E16	Improved epileptic impairments	[144]
MIA mice, 20 mg/kg poly(I:C) at E12.5	*n* = 7–15, analyzed at young adults	Anti-IL-6 antibody at E12.5	Prevents PPI, LI, improved exploratory and social behavior	[146]
MIA mice, 20 mg/kg poly(I:C) at E12.5	*n* = 15–20, analyzed at young adults	Probiotics at E0.5 to P21	Reduced IL-6 and IL-17a, prevented ASD-like behaviors, GABAergic neurons in PFC	[150]
MIA mice, 1 mg/kg LPS at E15.5 and E16.0	*n* = 5–12, analyzed at E16.0	Si-based agent at E13.5 to E16.0	Protect fetus from miscarriage, placenta from inflammation, improved expressions of *Il6*, *Hmox1*, and *Ptgs*	[30]
MIA mice, 20 mg/kg poly(I:C) at E12.5	*n* = 22–31, analyzed at P7	Si-based agent at E8.5 to P7	Improvements in mouse vocal communication, expressions of *Il6* and *Ifna1*	[31]
VPA mice, 600 mg/kg VPA at E12.5	*n* = 6–10, analyzed at young adults	HRW at E12.5 to P42	Impairments in ASD-like behaviors, pain sensation, anxiety-like behavior, memory, IL-6 and TNF-α	[163]
VPA mice, 600 mg/kg VPA at E12.5	*n* = 10, analyzed at young adults	Astaxanthin at P26 to P56	Improved ASD-like behaviors, oxidative stress such as advance protein oxidation product, nitric oxide, catalase, superoxide dismutase	[164]
VPA rats, 600 mg/kg VPA at E12.5	*n* = 7–12, analyzed at young adults	NAC at P23 for 4 weeks	Improved ASD-like behaviors, increased glutathione, reduced malondialdehyde	[165]
ASD model zebrafish, 600 µg/L fipronil and 600 µg/L pyriproxyfen	*n* = 15, analyzed at young adults	Vitamin C at P45 for 14 days	Impairments in social behaviors, lipid peroxidation, oxidative stress such as superoxide dismutase, glutathione peroxidase	[166]
VPA rats, 300 mg/kg VPA at P4 or 30 mg/kg VPA at P4 for 3 days	*n* = 7–16, analyzed at young adults	Methionine at P4 for 3 days	Improved ASD-like behaviors, expressions of antioxidant genes in PFC	[167]
VPA rats, 400 mg/kg VPA at P14	*n* = 12, analyzed at young adults	Green tea extract at P14 to P41	Improved ASD-like behaviors and Purkinje cells	[168]

MIA: maternal immune activation, LPS: lipopolysaccharide, VPA: valproic acid, HRW: hydrogen-rich water, NAC: *n*-acetyl L-cysteine, PFC: prefrontal cortex.

## 7. Hydrogen Medicine

The function of molecular hydrogen as an anti-inflammatory and antioxidant agent has attracted attention in both basic and clinical research fields (Figure 3). Medical hydrogen shows antioxidant, anti-inflammatory, antiallergic, and anti-apoptotic effects in both humans and animals [169,170,171,172]. Hydrogen selectively reduces hydroxyl radicals (•OH) in ROS, reacts only with hydroxyl radicals, and consequently can be used as a therapeutic agent without side effects for diseases associated with oxidative stress and inflammation in humans [173,174]. Hydrogen also has the advantage of being neutral, inexpensive, and permeable. 

The biological effects of hydrogen are manifold [170,175]. Hydrogen reduces oxidative damage, lipid oxidation, the levels of the oxidatively damaged base 8-OHdG, and gene mutations by scavenging hydroxyl radicals [170,175]. Hydrogen also reduces oxidative damage and protein nitrolation (-O-NO_2_) or nitrosolation (-S-NO_2_) by scavenging peroxynitrite (ONOO−) [171,176]. Hydrogen also reduces the levels of inflammatory cytokines involved in inflammation and oxidative stress, and increased NFE2L2 (NRF2) expression results in reduced oxidative stress in both humans and animals [30,31,170,175].

**Figure 4:**
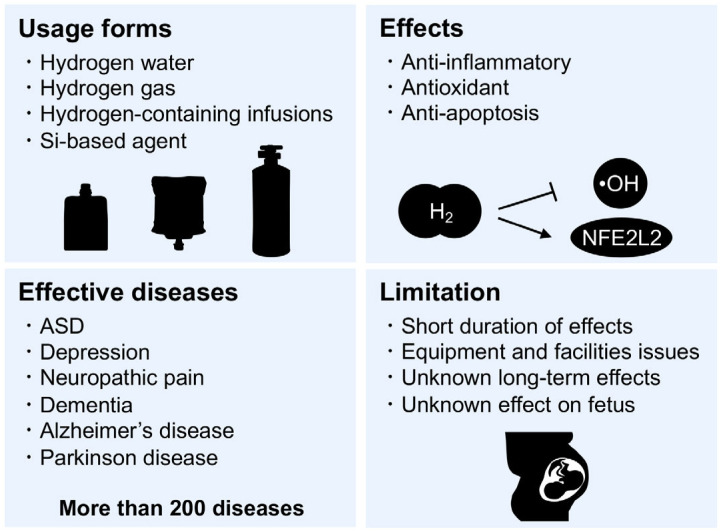
Clinical implications and limitation of hydrogen medicine. In hydrogen medicine, hydrogen gas, hydrogen water, and hydrogen-containing infusions are mainly used. It is used in a variety of ways, including therapeutic agents for diseases targeting inflammation and oxidative stress (as anti-inflammatory agents and antioxidants), protection and transplantation of organs and tissues. It has shown efficacy in more than 200 diseases, including cerebral ischemia, depression, and dementia. •OH: hydroxyl radicals, NFE2L2: as known as NRF2.

Hydrogen therapy can be delivered in several ways, including inhalation of hydrogen gas (HG), drinking hydrogen-rich water (HRW), injection of hydrogen-rich saline (HRS), hydrogen-containing infusions, taking a hydrogen bath, dropping HRS into the eyes, and increasing the production of intestinal hydrogen by bacteria [176]. The potential therapeutic effects of medical hydrogen for approximately 200 different diseases have been reported in human and animal models [175] (Figure 4). As medical hydrogen, HG and HRW are most commonly used in both clinical practice and research related to cerebral ischemia, dementia, Alzheimer’s disease, Parkinson disease, depression, spinal cord injury, neuropathic pain, diabetes, metabolic syndrome, cardiovascular disorders, hepatic injuries, mitochondrial diseases, and other similar conditions [170,171,175,176] (Figure 4).

As a note, prior research on hydrogen medicine solely focused on the protective effects of hydrogen; however, the effects of hydrogen therapy, especially long-term hydrogen intervention, on physiological function in a normal state remain largely unknown. The effects of long-term administration are of interest because the effects of hydrogen on physiological function under normal conditions have been largely ignored. Long-term administration study of HG and HRW in rats showed no apparent adverse effects, but long-term administration has been reported to change various biochemical characteristics, such as body weight, serum lipids, liver function, and myocardial function markers [177]. In addition, more careful consideration is needed regarding the effects of such subtle changes on pregnancy.

## 8. Si-Based Hydrogen-Producing Agent

In this section, we discuss potential novel therapeutic agents that target both inflammation and oxidative stress. Using the principles of hydrogen medicine, we have recently developed a Si-based hydrogen-producing agent (Si-based agent) that can continually produce large amounts of hydrogen (up to 400 mL/g) by reaction with water under conditions (pH 8.3 and 36 °C) similar to those in the gut [27,28,29]. Si and its reaction product SiO_2_ are nontoxic, enabling the oral administration of this Si-based agent. The protective effects of enteric hydrogen generated from Si-based agent have been reported in animal models of maternal-fetal transmission and MIA [30,31]. Since the hydrogen is produced in the gastrointestinal system, it is physically and easily delivered to the uterus and fetus. Therefore, our Si-based agent may prevent health risks related to inflammation and oxidative stress. 

In our previous study, we demonstrated that social communication in MIA-affected mouse offspring used as an ASD model was improved by a Si-based agent [31]. In general, MIA offspring mice are used as ASD model mice, resulting in reductions of the number of ultrasonic vocalizations (USVs) at postnatal stages [31,38,178,179,180]. However, when pregnant mice received preventive treatment with a Si-based agent before induction of MIA, the number of declined USVs in the MIA mouse offspring was recovered [31]. Si-based agent also inhibits the expression of inflammatory genes such as *Ifna1* and *Il6* in the PFC of MIA mice [31], suggesting that a Si-based agent is a potential drug to protect the fetus from MIA-related inflammation in ASD.

To further investigate the neuroprotective effects of our Si-based agent, we analyzed a mouse model of mother-to-child transmission. Mother-to-child transmission of viruses and bacteria increases the risk of miscarriage and various diseases in children [181,182]. Such transmissions can result in infections and diseases in infants or in the induction of an inflammatory immune response through the placenta in humans [181,182]. Pregnant mice treated with a Si-based agent showed lower rates of miscarriage due to mother-to-child transmission and suppression of inflammation and neutrophil infiltration in the placenta [30]. The Si-based agent also suppresses *Il6* expression in the mouse placenta and induces the expression of antioxidant and anti-apoptotic genes such as *Hmox1* and *Ptgs2* [30], suggesting that the Si-based agent protects the fetus from the inflammation associated with mother-to-child transmission.

Our findings require further verification, but the results demonstrate that a Si-based agent can protect the fetus from MIA and mother-to-child transmission by suppressing inflammation in mice, suggesting that a Si-based agent may be useful as a prophylactic agent for ASD caused by MIA. Therefore, we plan to verify the efficacy of the Si-based agent in various ASD model animals, and validate its safety in pregnant mice and their offspring. Therefore, while we have not reached a debate on whether hydrogen medicine is beneficial in the treatment of ASD, results suggest a preventive effect have been obtained in MIA animal models [31].

## 9. Preclinical Studies of Antioxidants in ASD Model Animals

In the preclinical studies of ASD, antioxidant effects have been reported using ASD model animals (Table 2). Valproic acid (VPA) exposure during pregnancy increases the risk of ASD in offspring (VPA mice or rats), which has been widely used as a method for producing ASD model animals [10,55]. In VPA-induced ASD model mice, HRW administration improved ASD-like behaviors such as social behavior and pain sensation as well as anxiety-like behavior and memory impairment and IL-6 and TNF-α inflammatory cytokines [183]. 

In addition, it has been reported that administration of the antioxidant astaxanthin to improve behavioral abnormalities in social and anxiety-like behaviors and oxidative stress such as advance protein oxidation product, nitric oxide, catalase, and superoxide dismutase in VPA-induced ASD model mice [163]. Administration of antioxidant N-acetyl L-cysteine (NAC), a precursor of cysteine, is known to boost the synthesis of intracellular glutathione [164,184]. It Is reported that repetitive behaviors and oxidative stress in VPA-induced ASD model rats were improved by increasing glutathione and reducing malondialdehyde induced by NAC administration [185]. Vitamin C is a potent antioxidant that can neutralize and remove oxidants, such as highly reactive molecules generated in metabolic processes in the brain but also in other organs in humans and animals [165,186,187]. In ASD model zebrafish exposed to a mixture of fipronil and pyriproxyfen, it has been reported that impairments of social behaviors and increases in oxidative stress such as superoxide dismutase, glutathione peroxidase, and lipid peroxidation were improved by vitamin C [188]. It has been reported that a treatment with methyl donor methionine improved ASD-like behaviors and the expressions of antioxidant genes in the PFC of VPA-induced ASD model mice [166]. Administration of green tea extract to VPA-induced ASD model mice has also been reported to improve ASD-like behaviors and impairment of Purkinje cells [167].

These studies demonstrate that antioxidants improve ASD-like phenotypes such as oxidative stress and inflammation, behavioral symptoms, and neurodevelopmental abnormalities in ASD animal models.

## 10. Clinical Studies of Antioxidants in ASD

In the clinical studies, the studies have reported the effects of antioxidants on ASD (Table 1). By comprehensive metabolome analysis, oxidative stress, mitochondrial dysfunction, elevated hormone levels, altered lipid profiles, and altered levels of phenolic microbial metabolites have been reported in the serum and feces of children with ASD [168]. We have also reported an increased oxidative stress in children with ASD [121,122,123]. In addition, specific metabolite levels to cysteine, methionine, and glutathione pathways were correlated with autism diagnostic observation schedule (ADOS) scores of ASD symptoms [168]. 

Clinical studies have reported the effects of antioxidants against oxidative stress in ASD. In a double-blind, placebo-controlled study, vitamin C supplementation in children with ASD improved sensorimotor behaviors [189,190]. Coenzyme Q10 is a mitochondrial antioxidant cofactor that crosses the BBB. Administration of ubiquinol (the reduced form of coenzyme Q10) has been reported to improve communication in children with ASD [155]. Coenzyme Q10 supplementation was reported to reduce oxidative stress and ASD symptoms in children with ASD [156]. In a double-blind, placebo-controlled trial, administration of the broccoli-derived antioxidant sulforaphane improved social interaction and communication in adults with ASD [157]. A randomized, placebo-controlled trial reported improvement in hypersensitivity subscales with administration of the antioxidant N-acetylcysteine (NAC) in children with ASD [158]. In a randomized, double-blind, placebo-controlled trial, the combination of risperidone and NAC reduced hypersensitivity subscales in children with ASD [159,160]. Additionally, a randomized, double-blind, placebo-controlled clinical trial reported that the combination of risperidone and NAC reduced hypersensitivity in children with ASD [191]. Moreover, high doses of cocoa, which has antioxidant properties, improved social communication, erratic behavior, and self-regulatory behavior in children with ASD [161]. In randomized, double-blind, placebo-controlled trials, melatonin treatment, acting as a potent antioxidant, improved daytime behavior in ASD patients [162].

These clinical studies indicate that excessive oxidative stress in ASD is the cause of exacerbating pathology and symptoms, and that the reduction of oxidative stress using antioxidants improve the symptoms of ASD.

## 11. Conclusions

In this review, we discussed neuroinflammation and oxidative stress as risk factors in the pathogenesis of ASD. The prenatal environment is not only a sensitive period for fetal development but also a vulnerable period for the onset of disorders such as NDDs and psychiatric disorders [9,10,11,192]. MIA, one of the environmental factors involved in the onset of ASD, induces neuroinflammation and oxidative stress in the offspring and directly or indirectly damages the placenta and fetal brain during embryonic stages (Figure 1). In addition, increased oxidative stress in the offspring caused by MIA or other factors has also been reported to be involved in the pathogenesis of ASD (Figure 2). Therefore, as a future direction of defense strategies targeting neuroinflammation and oxidative stress underlying pathogenesis of ASD, anti-inflammatory agents and/or antioxidants may be viable options (Figure 3 and Figure 4). However, it has not been proven that the increased oxidative stress in MIA was directly induced by inflammation and may be a secondary aspect resulting from inflammation. Future research needs to clarify this point.

On the other hand, the mother’s body is under the special immune environment during pregnancy, that differs from normal condition, and the maternal immune system is highly engaged in the successful progression of pregnancy from implantation to delivery [16]. For example, IL-6 is upregulated in utero during normal implantation [193]. Oxidative stress is also essential for maintaining normal physiological functions in the development, and plays an essential role during pregnancy [194,195,196]. Thus, the effects of long-term administration of anti-inflammatory agents and/or antioxidants during pregnancy should be considered more carefully. In fact, long-term administration of HG and HRW in rats has reported that it caused the change in body weight, metabolism, and biochemical serum markers [177]. The long-term effects corresponding to the gestational age of humans are difficult to observe in animal models such as mice and rats. At the end, we hope that a treatment method using anti-inflammatory agents and/or antioxidants for ASD and NDDs targeting pregnancy and the neonatal period will be established in the near future.

## Data Availability

Not applicable.
